# Music Therapy for Depression Enhanced With Listening Homework and Slow Paced Breathing: A Randomised Controlled Trial

**DOI:** 10.3389/fpsyg.2021.613821

**Published:** 2021-02-16

**Authors:** Jaakko Erkkilä, Olivier Brabant, Martin Hartmann, Anastasios Mavrolampados, Esa Ala-Ruona, Nerdinga Snape, Suvi Saarikallio, Christian Gold

**Affiliations:** ^1^Faculty of Information Technology, University of Jyväskylä, Jyväskylä, Finland; ^2^NORCE Norwegian Research Centre AS, Bergen, Norway; ^3^Department of Clinical and Health Psychology, Faculty of Psychology, University of Vienna, Vienna, Austria

**Keywords:** depression, anxiety, music therapy, randomised controlled trial, resonance frequency breathing, homework

## Abstract

**Introduction:** There is evidence from earlier trials for the efficacy of music therapy in the treatment of depression among working-age people. Starting therapy sessions with relaxation and revisiting therapeutic themes outside therapy have been deemed promising for outcome enhancement. However, previous music therapy trials have not investigated this issue.

**Objective:** To investigate the efficacy of two enhancers, resonance frequency breathing (RFB) and listening homework (LH), when combined with an established music therapy model (trial registration number ISRCTN11618310).

**Methods:** In a 2 × 2 factorial randomised controlled trial, working-age individuals with depression were allocated into groups based on four conditions derived from either the presence or absence of two enhancers (RFB and LH). All received music therapy over 6 weeks. Outcomes were observed at 6 weeks and 6 months. The primary outcome was the Montgomery Åsberg Depression Rating Scale (MADRS) score.

**Results:** There was a significant overall effect of treatment for the primary outcome favouring the breathing group (*d* = 0.50, 95% CI 0.07 to 0.93, *p* = 0.02). The effect was larger after adjustment for potential confounders (*d* = 0.62, 95% CI 0.16 to 1.08, *p* = 0.009). Treatment effects for secondary outcomes, including anxiety (anxiety scale of Hospital Anxiety and Depression Scale) and quality of life (RAND-36), were also significant, favouring the breathing group. The homework enhancer did not reach significant treatment effects.

**Conclusion:** We found that the addition of RFB to a music therapy intervention resulted in enhanced therapeutic outcome for clients with depression.

## Introduction

### Impact of Depression

Depression is one of the most disabling of diseases, causing a serious individual and societal burden (Sobocki et al., 2006). In Europe, major depression and specific phobia are the most common psychiatric disorders (Alonso et al., 2004). Almost 13% of the population report a lifetime history of major depressive disorder, with around 4% having experienced major depression in the past 12 months. Depression is often connected to other disabling disorders, such as generalised anxiety disorder and somatoform disorder, all of which show an excess comorbidity leading to higher psychosocial disability, increased suicidality, and worse clinical outcome and treatment response (Maier and Falkai, 1999). According to Turunen (2020), the prevalence of mental problems in Finland has been growing continuously in recent years; the number of anxiety diagnoses, for instance, was 25% higher in 2019 compared to the year before. Also the effect of COVID-19 can be clearly seen in the use of mental health services, the number of short-term psychotherapy referrals across countries having increased four times at the beginning of 2020, compared to the same period one year before (Khan et al., 2020). In the light of these trends, offering the best possible evidence-based treatments and improving existing therapeutic approaches has become more important than ever. The aim of this study was to investigate whether an effective form of music therapy could be further enhanced in terms of clinical outcomes.

### Treatments for Depression

Pharmacotherapy and psychotherapy—used alone or in combination—are currently the main treatments for depression (Masennus: Käypä hoito-suositus, 2020), and both have been found equally efficacious (De Maat et al., 2006). However, when including risk of relapse, long-term outcomes, and suicidal risks in the evaluation, pharmacotherapy has been associated with higher relapse (De Maat et al., 2006), poorer long-term outcomes (Hengartner et al., 2018), and increased suicidal risks (Baldessarini et al., 2017), making psychotherapy an appealing and valuable option among the treatment modalities. Interestingly, a recent meta-analysis (Weitz et al., 2018) reports that psychotherapy is almost as effective at reducing comorbid anxiety symptoms as it is at reducing depressive symptoms. Furthermore, besides the reduction in depressive symptoms, psychotherapy also has a positive impact on quality of life (QoL), especially its mental health component (Kolovos et al., 2016).

### Forms of Psychotherapy

When comparing the most common forms of verbal psychotherapy used in the treatment of depression, Cuijpers et al. (2014) found no significant difference in terms of response and remission rate, which suggests that the various forms of verbal psychotherapy might be largely interchangeable. A common challenge for verbal psychotherapy is the fact that major depression typically leads to psychomotor regression in the area of speech (Flint et al., 1993), noticeable in the form of retardation of speech and prolongation of quiet episodes (Hoffman et al., 1985). Consequently, verbal expression and processing during therapy may be difficult or insufficient for some individuals with depression. Psychotherapy forms that allow non-verbal expression – such as arts therapies – may offer a potential alternative. For instance, there is an increasing number of randomised controlled trials (RCT) and two Cochrane systematic reviews (Maratos et al., 2008; Aalbers et al., 2017) on the effect of music therapy for depression. According to Aalbers et al. (2017), music therapy provides short-term beneficial effects for people with depression. More specifically, music therapy added to treatment as usual (TAU) appears to be more efficacious than TAU alone. Furthermore, music therapy is not associated with more or fewer adverse events than TAU alone. Similarly, a systematic review on the effectiveness of dance and movement therapy (DMT) in the treatment of adults with depression also concludes that DMT is an effective intervention (Karkou et al., 2019).

### Improvisational Music Therapy

We previously conducted an RCT on the effectiveness of music therapy for working-age people with depression (Erkkilä et al., 2011). In that trial, only one specific music therapy technique was used, called improvisational psychodynamic music therapy (IPMT) (Erkkilä et al., 2012). This decision was influenced by the first systematic review on music therapy for depression (Maratos et al., 2008), which concluded that one weakness of the existing RCTs was the variety of music therapy methods included in the same study, making it difficult to draw any conclusions on the effect of a single method, such as clinical improvisation. In that RCT, based on 20 bi-weekly music therapy sessions of 60 min each, we found that the clients in the IPMT + TAU group improved significantly more in terms of depression, anxiety, and general functioning, compared to the TAU group. Furthermore, the treatment response of the IPMT + TAU group was almost twice as high as in the TAU group, based on the primary outcome measure (depression). We concluded that IPMT is an effective treatment for depression when added to TAU, with the added benefit of significantly reducing comorbid anxiety and improving general functioning. The core element of IPMT, free improvisation, can be described as a means of “self-projection and free association” and may enable clients thereby “to connect with emotional memories and images” (Erkkilä et al., 2011, p. 132). Emphasising the creative process rather than the end product, it has also been described as “playing around with sounds until they form whatever patterns, shapes or textures one wants them to have, or until they mean whatever one wants them to mean” (Bruscia, 1998, p. 5). In the present study, we aimed to build on the positive results of our previous RCT, and investigate whether the effectiveness of integrative improvisational music therapy (IIMT; based on IPMT with certain modifications, as described in “Methods”) can be further enhanced through the addition of carefully selected elements. The two elements we chose were a slow-breathing technique called resonance frequency breathing (RFB), and a homework task where clients were encouraged to listen to the improvisations created during therapy.

### Enhancement 1: Resonance frequency breathing (RFB)

Resonance frequency breathing is the core element of a method called heart rate variability biofeedback (HRVB). With the help of biofeedback equipment displaying heart and respiration patterns in real-time, clients learn to breathe at their resonance frequency, which corresponds to a specific breathing speed that is unique to each person, and is typically located between 4.5 and 6.5 breaths/min in adults (Vaschillo et al., 2006). When breathing at resonance frequency, heart, respiratory, and blood pressure rhythms become highly synchronised, and heart rate variability (HRV) substantially increases (Lehrer and Gevirtz, 2014). Within a very short time, the autonomic nervous system shifts to parasympathetic dominance (rest-and-digest), resulting in relaxation and lower stress levels. RFB is a simplified form of HRVB, as it does not involve any biofeedback equipment. In RFB, the resonance frequency is determined beforehand through a single breathing assessment. Subsequently, clients are doing paced breathing at their previously determined resonance frequency, using a breath pacer set at the right speed, according to the results of the breathing assessment. In terms of application, HRVB has proven beneficial for a wide range of physical and psychological conditions (Gevirtz, 2013; Moss and Shaffer, 2017), as well as for the enhancement of artistic creativity (Gruzelier et al., 2014) and sport performance (Jiménez Morgan and Molina Mora, 2017). More relevant to the topic of the present trial, a recent meta-analysis, based on 24 studies and 484 participants, revealed that HRVB was associated with a large reduction in stress and anxiety (Goessl et al., 2017). HRVB has also been found beneficial for the treatment of depression, both in open-label studies (Karavidas et al., 2007; Siepmann et al., 2008) and in controlled studies (Caldwell and Steffen, 2018; Lin et al., 2019). In a systematic review and meta-analysis investigating the effect sizes of HRVB for specific health conditions, the authors conclude that HRVB would be a useful addition to clinicians’ existing skill-sets, because of its proven efficacy and the ease with which it can be used alongside other forms of therapy (Lehrer et al., 2020). However, to date, very few attempts have been made to fully integrate HRVB into an existing form of (psycho)therapy, so as to create a synergy effect in support of the latter. In most studies we have come across, HRVB is used as an additional and separate treatment modality, for example alongside cognitive behavioural therapy or acceptance and commitment therapy (Reiner, 2008; Caldwell and Steffen, 2018). At the Music Therapy Clinic for Research and Training (University of Jyväskylä, Finland), we have developed and tested our own therapy format, whereby each session of IIMT begins with 10 min of RFB. Our pilot studies suggest that the inclusion of RFB helps clients upregulate and downregulate their emotions during music therapy, depending on their clinical status and current needs (Brabant and Erkkilä, 2018). These preliminary findings require follow-up with a between-group study such as the present one, to determine whether the observed effects on therapy processes also lead to better outcomes. Generally, it should be noted that RFB is an active field of research. The mechanisms behind RFB are incompletely understood, but may include baroreflex gains (Shaffer and Meehan, 2020); vagal nerve stimulation (Gerritsen and Band, 2018); enhancement of functional connectivity in brain areas associated with emotion regulation (Mather and Thayer, 2018); and the complex interplay of several neurophysiological processes (Noble and Hochman, 2019). However, there is consensus that the resonance frequency is stable in adults, around 0.1 Hz or 6 bpm, and that breathing at a frequency near 0.1 Hz promotes relaxation and other physical and mental benefits (Mather and Thayer, 2018; Noble and Hochman, 2019; Shaffer and Meehan, 2020). Slow-placed breathing may provide a parsimonious explanation of the physical and mental benefits of a number of contemplative activities such as meditation or yoga (Gerritsen and Band, 2018), but it is less clear whether breathing at the individual’s precise resonance frequency is more effective than breathing at 6 bpm (Shaffer and Meehan, 2020). Procedures for frequency assessment have been reviewed recently (Shaffer and Meehan, 2020), based on previous work by Lehrer and colleagues (Lehrer and Gevirtz, 2014; Lehrer et al., 2020).

### Enhancement 2: Listening homework (LH)

The idea of the LH task arose from our earlier clinical observations, where some clients seemed to benefit from listening back to the recorded music improvisations, both during the sessions and at home. We hypothesise that, because music improvisations evoke emotions and imagery with specific therapeutic meanings, providing clients with the chance to further process these emotions at home may improve the effect of therapy. The therapeutic potential of homework is already known in the context of verbal psychotherapy (Kazantzis et al., 2000; Kazantzis et al., 2010; Mausbach et al., 2010), where it has been used for the treatment of depression (Thase and Callan, 2006). According to the meta-analysis by Mausbach et al. (2010), clients’ compliance to homework is a crucial factor, with higher compliance being associated with better therapeutic outcomes. While this body of research supports the plausibility of homework in psychotherapy in general, it is not directly related to LH in this study. First, the previous research involved predominantly cognitive and behavioural therapy (CBT), which is quite distant from IIMT. Second, LH is rather different from the types of homework assignments typically given in these other types of psychotherapies. However, the idea of LH is closely connected to a category of music therapy methods called receptive music therapy. In receptive music therapy, listening to music is used to stimulate the verbal dialogue between client and therapist, and to evoke emotions, memories, images, associations, and so on. The music is often precomposed, but can also be improvised by a therapist in a given situation. In this context, music is often seen as a catalyst and enhancer. In one of the best-known examples of receptive methods – the Bonny Method of Guided Imagery and Music (BMGIM) (Grocke and Bruscia, 2002)– pre-designed programmes of Western classical music are used to shape and support the client in experiencing unfolding imagery. The client listens to the programme while in an altered state of consciousness and simultaneously dialogues with the therapist. From a therapeutic perspective, the BMGIM approach and the experiences in altered state as an essential element of it have been found beneficial and effective (Hammer, 1996; McKinney et al., 1997; McKinney and Honig, 2017). In contrast to BMGIM, however, in our study there was no therapeutic guidance *during* the home listening, although there were opportunities to discuss the listening experiences when being back in the therapy room.

### Hypotheses

In this RCT, we examined two hypotheses concerning the efficacy of RFB and LH when combined with IIMT to enhance therapeutic outcome. Hypothesis 1 suggested that RFB would reduce depressive symptoms and that we would observe a significant overall treatment effect over time for RFB, together with significant treatment effects post-intervention and at follow-up. Hypothesis 2 suggested that LH would similarly reduce depressive symptoms and yield significant treatment effects. These hypotheses are rationalised by the aforementioned findings, which indicate positive treatment effects of both HRVB and psychotherapeutic homework assignments in depressed clients. In addition to this, we were interested in exploring potential interaction effects between the RFB and LH interventions, although due to insufficient literature we did not have an *a priori* hypothesis on the efficacy of this combination for the treatment of depression.

## Materials and Methods

### Design

We conducted a 2 × 2 factorial randomised controlled trial in which all clients received IIMT (Erkkilä et al., 2019). The trial was registered (ISRCTN11618310) before recruitment. Clients were randomly allocated to one of four groups (IIMT alone, IIMT + LH, IIMT + RFB, IIMT + LH + RFB) following a 2 × 2 factorial design. Conditions were derived from either the presence or absence of LH (LH_yes_, LH_no_) and RFB (RFB_yes_, RFB_no_).

### Participants

Eligible participants were adults with a primary diagnosis of major depressive disorder (F32/F33, ICD-10 criteria). The diagnosis was made by a psychiatric nurse with an MA degree in nursing science and assessment qualification. Musical skills were not required from participants. Exclusion criteria were a known history of psychosis, bipolar disorder, personality disorder, other combined psychiatric disorders in which depression cannot be defined as primary disorder, acute and severe substance misuse, and depression severity impeding clinical measurements or verbal conversation.

### Randomisation and Blinding

After screening and diagnosis, a computerised block randomisation with randomly varying block sizes of 4 and 8 was conducted by an external person (C.G.) who had no direct contact with the patients. To ensure group allocation concealment, randomisation was conducted at another site (NORCE Norwegian Research Centre). Thus, assessor, therapists, and participants were unaware of allocation until therapy started. As this was a single-blind trial, only the outcome assessor remained blinded to allocation throughout the trial.

### Assessment Procedure

Outcome measures were collected by a specialist in psychiatric assessment at three measurement points: (1) baseline, i.e., during recruitment (T0); (2) post-intervention, i.e., 6 weeks after randomisation (T1); (3) and follow-up, i.e., 6 months after randomisation (T2). The time point of primary interest was post-intervention. Demographic information was obtained at the beginning of the intervention.

### Interventions

All participants were offered 12 bi-weekly sessions of IIMT over a period of 6 weeks. Each session lasted one hour. The therapeutic approach and its additional components (LH and RFB) are described in the following sections.

#### Integrative Improvisational Music Therapy (IIMT)

In music therapy, music experiences are used to enrich and enhance a client’s expression and interaction. Essential to music therapy is the client-therapist relationship, in contrast with music and medicine, where music can be used without that relationship. IIMT, developed at the Music Therapy Clinic for Research and Training (University of Jyväskylä, Finland), is based on clinical improvisation, which is one of the major methods of music therapy (Bruscia, 1987). IIMT is based on the interplay and alternation between free music improvisation and verbal discussion (Erkkilä et al., 2011, 2012). It was originally anchored in the psychodynamic music therapy tradition (Priestley, 1994; Bruscia, 1998), and later on, adopted elements from the integrative psychotherapy tradition (Norcross and Goldfried, 2005) as well. The fundamental aim of IIMT is to encourage clients to engage in expressive musical interaction with the therapist. The experiences arising from this interaction are then conceptualised and further processed in the verbal domain (Erkkilä et al., 2011). In IIMT, improvising is primarily understood both as a symbolic representation of abstract mental content, and as an expressive medium able to evoke emotions, images, and memories (Erkkilä et al., 2012), but other human processes–such as cognitive, behavioural, and physiological–may be involved as well.

We standardised the clinical setting so that every therapy process involved identical instruments and a similar arrangement of the two music therapy clinics. Two identical digital pianos placed opposite each other (one for the client, another one for the therapist) were used for melodic and harmonic improvisations. Two identical djembe drums placed next to the pianos were used for non-melodic, rhythmic improvisations. No other instruments or music therapy methods were used. The improvisations were digitally recorded, which made it possible to listen back to them anytime afterwards. Eleven qualified and clinically experienced music therapists (five female, six male) were responsible for conducting the therapy sessions.

#### Added Component: Resonance Frequency Breathing (RFB)

Each client’s resonance frequency was determined through a breathing assessment conducted before the beginning of therapy. We opted for a single assessment for the sake of simplicity, relying on the finding that adults’ resonance frequency appears to be very stable (Vaschillo et al., 2006). The assessment followed the protocol developed by Lehrer (2007), and consisted of two parts. First, the client was instructed in how to perform RFB (abdominal breathing, inhalation through the nose and exhalation through the mouth, no holds or pauses, and breathing slower without breathing deeper). Once the technique was sufficiently mastered, the client was asked to breathe at six different rates for 3 min each, while wearing a heart rate monitor. The breathing rates ranged from 7 to 4.5 breaths/min, starting from the fastest until the slowest, in 0.5 steps. Heart rate data for each breathing segment was then analysed using Kubios HRV 3.1 (Tarvainen et al., 2014). The optimal breathing rate was defined as the rate producing the highest peak in the low frequency (LF) component of the power spectrum (0.04–0.15 Hz), as obtained through a fast Fourier transform analysis of the heart beat intervals.

Following the assessment, each client’s optimal breathing speed was communicated to their respective therapist, who used this information for the RFB task. At the beginning of each therapy session, clients assigned to RFB_yes_ performed 10 min of RFB at an inhalation/exhalation ratio of 40/60 in a seated position, while following visual cues provided by a breathing app called Kardia (Tache, 2017), installed on a tablet computer placed in front of the client. Longer exhalations are known to promote parasympathetic activation (Strauss-Blasche et al., 2000) and, in a slow-breathing scenario, a 40/60 ratio has been shown to induce higher levels of relaxation than its opposite ratio (Diest et al., 2014).

#### Added Component: Listening Homework (LH)

Listening homework was conducted outside the therapy context, in the client’s own time, based on the clinical improvisations created in music therapy sessions using two digital pianos and two djembes. These improvisations were recorded by the therapists using Pro Tools 11.3.1. Each client had personal access through their personal computers to all of their improvisation recordings. Recordings were stored on a University server and automatically synchronized with the clients’ home computers using the continuous file synchronization program Syncthing (The Syncthing Foundation, 2017) in order to be available for listening immediately after the music therapy session. All improvisations created during the music therapy process were available to the client for listening throughout the therapy process. Clients were instructed to use headphones to listen, whenever they felt like doing so and as many times as they wished, to any of the available improvisations and could decide when and how many times they wanted to listen to the improvisations. A dedicated music player, Cantata (2017), which automatically displayed all available improvisations to clients, was installed in clients’ computers for this purpose. Software installation and guidance to clients on how to use the music player was performed shortly before the first music therapy session. Client’s mean total listening time was 02h:28m:59s (SD = 03:03:34; median = 01:10:32; Q1 = 00:26:20; Q3 = 03:34:29; range 0 to 12:11:21).

At the beginning of the trial, the clinicians were advised to encourage clients to listen to the improvisations after each session. In addition, the therapists were advised to recommend particular improvisations to be listened to at home when they were connected to specific, clinically important themes. Clients’ experiences while listening back to improvisations could be discussed and reflected upon with the therapist in subsequent therapy sessions.

### Treatment Fidelity

To ensure treatment fidelity, the selected clinicians were offered intensive training in the music therapy model and in the two added components. All the clinicians were qualified music therapists. Regular clinical supervision was used for monitoring and maintaining the quality of the clinical work.

### Outcomes

#### Primary Outcome

The Montgomery-Åsberg Depression Rating Scale (MADRS) (Montgomery and Åsberg, 1979) was the primary outcome of the study. At the beginning of the study, MADRS was used to determine participant eligibility. The MADRS has high joint-reliability, has been shown to be sensitive to change, and has been demonstrated to have predictive validity for major depressive disorder (Rush et al., 2008).

#### Secondary Outcomes

The anxiety subscale (HADS-A) of the Hospital Anxiety and Depression Scale (HADS) (Aro et al., 2004) was used to assess anxiety. QoL was assessed using the RAND-36 (Aalto et al., 1999), whose results were aggregated into two summary scales, physical component sum (PCS) and mental component sum (MCS) (Ware and Kosinski, 1994). A detailed explanation of this procedure can be found in the Supplementary Material. The Global Assessment of Functioning (GAF) (Jones et al., 1995) was used for assessing how mental health symptoms affected the clients’ daily life and general functioning. The measures of general functioning and QoL were chosen based on widespread use in psychological intervention studies concerning people with mental health problems.

### Sample Size

Following a previous IIMT intervention, we assumed that no more than 10% of clients would leave the study early. We aimed to recruit 68 participants and allocate them into 4 conditions in a factorial design (*n* = 34 in each condition; *n* = 17 in each group) (Erkkilä et al., 2019). For each condition, the selected sample size provided statistical power of 0.80 for detecting a medium standardised effect size of Cohen’s *d* = 0.60 in a mixed-model analysis (see Twisk, 2013, p. 281, equation 13.3), with a 2-tailed significance level of *p* < 0.05 and intra-participant correlation of ρ = 0.6.

### Statistical Analysis

An intention-to-treat (ITT) approach was followed, using all available data regardless of whether the treatment was received as intended. Clients who left the study before completion of the intervention were considered dropouts. All tests used two-tailed 5% significance level, with no adjustments for multiplicity. Baseline, post-intervention and follow-up outcome measures served as continuous dependent variables. Repeated-measures linear mixed-effects models (see Supplementary Material) were used to assess RFB and LH effects for each continuous outcome. An advantage of the utilised repeated measures design is that clients with missing data can be retained in the model, and thus all clients were used in the analysis. RFB and LH were entered as predictors and a random intercept term grouped by client was added to adjust for the dependency of repeated observations within each client. To adjust for baseline differences between conditions, the treatment terms were removed from the model (Twisk et al., 2018). Hence, the effects of RFB and LH were calculated from the interaction between each factor and time. As an exploratory investigation to examine potential interaction effects between RFB and LH interventions, the repeated-measures linear mixed-effects models were subsequently expanded by adding an RFB x LH interaction.

Besides treatment effect post-intervention and follow-up, we obtained an overall treatment effect over time *B* as an estimate of the raw mean difference between presence and absence of each factor; *B* was calculated as the sum of the regression coefficients between each condition and time points (Erkkilä et al., 2019). To estimate effect sizes for a given outcome, its overall treatment effect over time was divided by the standard deviation of the measure across all clients at baseline.

For each client, a dichotomous treatment response variable was calculated, defined as a reduction in MADRS of at least 50% between the pre- and post-intervention measurements. For dichotomous variables (leaving the study early, treatment response), missing data were imputed and a negative outcome was assumed for those clients (left the study early, no-response) for a conservative estimate. Fisher’s exact test and odds ratio were calculated separately for RFB and LH. To determine clinical significance, risk difference and number needed to treat (NNT) were calculated for effects that were statistically significant.

Besides the crude efficacy analysis, an adjusted efficacy analysis and two sensitivity analyses were carried out. The repeated-measures linear mixed-effects model of each continuous outcome was adjusted for prognostic covariates by adding them as random effects (random slopes) in the model: age group (i.e., grouped every 10 years), gender, medication (use of antidepressants, anxiolytic or hypnotic medication), and therapist. Two sensitivity analyses were conducted for the primary outcome: a single imputation method (Last Observation Carried Forward) that assumes no change for missing data, and a per-protocol approach (treatment as received). All statistical analyses were performed in Matlab 2019b (MathWorks, Natick, Massachusetts).

### Data Sharing

The study’s data-set, except for the data that could compromise the privacy of research participants, is available from the corresponding author upon request.

## Results

**FIGURE 1:**
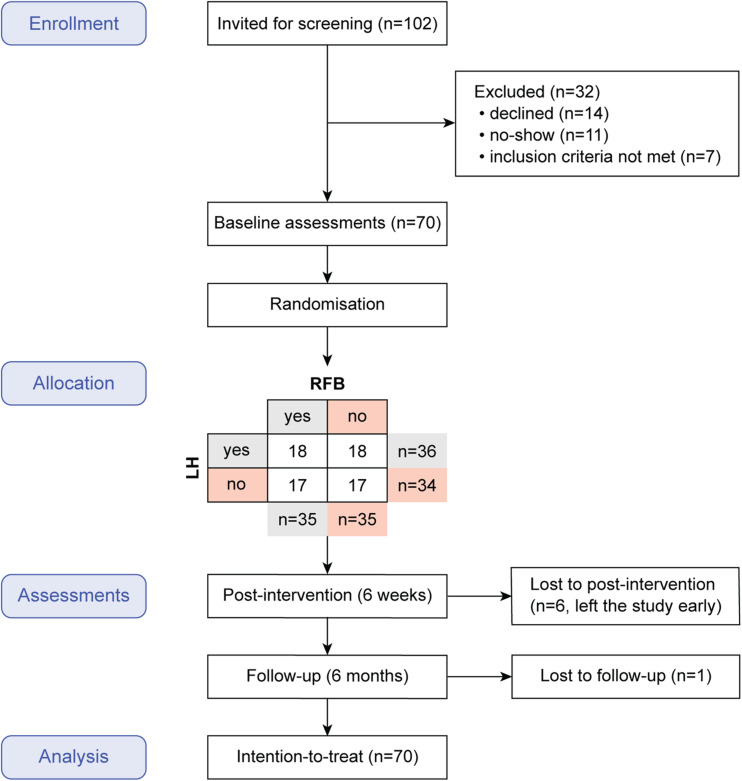
Flow of participants through the trial.

The study was conducted at the Music Therapy Clinic for Research and Training (University of Jyväskylä, Finland). Figure 1 shows the patient flow during the trial.

Recruitment started on February 1, 2018 and ended on October 31, 2018. Participants were recruited in central Finland through newspaper announcements. Of 102 people who were initially invited for screening, 14 declined, 11 were no-shows and 7 met an exclusion criterion. This left 70 eligible participants (74% female), their age ranging from 19 to 57 years (*M* = 39). Baseline characteristics in each condition are shown in Table 1.

**TABLE 1:** Demographic and clinical characteristics of 70 clients at baseline.

	RFB no (n = 35)		RFB yes (n = 35)		LH no (n = 34)		LH yes (n = 36)	
	Mean [SD]	Range	Mean [SD]	Range	Mean [SD]	Range	Mean [SD]	Range
Age, y	38.37 [11.44]	19 to 57	39.69 [11.59]	20 to 56	40.50 [12.75]	19 to 56	37.64 [10.07]	21 to 57
Female, n (%)	22 (62.86)	–	30 (85.71)	–	28 (82.35)	–	24 (66.67)	–
F32 diag., n (%)	29 (82.86)	–	24 (68.57)	–	24 (70.59)	–	29 (80.56)	–
F33 diag., n (%)	6 (17.14)	–	11 (31.43)	–	10 (29.41)	–	7 (19.44)	–
Medication, n (%)	18 (51.43)	–	17 (48.57)	–	17 (50)	–	18 (50)	–
MADRS	22.80 [7.38]	10.00 to 41.00	26.71 [6.18]	16.00 to 43.00	25.32 [6.95]	11.00 to 41.00	24.22 [7.17]	10.00 to 43.00
HADS	9.63 [4.22]	1.00 to 17.00	11.57 [3.30]	2.00 to 17.00	11.41 [3.88]	2.00 to 17.00	9.83 [3.79]	1.00 to 17.00
RAND-36 MCS	−5.51 [2.53]	−9.60 to 2.08	−6.33 [2.48]	−10.68 to −0.59	−6.12 [2.51]	−10.49 to −0.29	−5.74 [2.55]	−10.68 to 2.08
RAND-36 PCS	−3.59 [2.97]	−10.22 to 2.34	−3.71 [2.72]	−8.66 to 1.26	−4.07 [2.91]	−10.22 to 2.34	−3.26 [2.73]	−8.66 to 1.26
GAF	60.20 [7.77]	42.00 to 70.00	56.69 [8.23]	42.00 to 71.00	57.71 [8.82]	42.00 to 70.00	59.14 [7.50]	42.00 to 71.00

*RFB: resonance frequency breathing. LH: listening homework. SD: standard deviation. y: years. Total *n*: 70; RFB_no_*n*: 35; RFB_yes_*n*: 35; LH_no_*n*: 34; LH_yes_*n*: 36.*

According to the results of the treatment effect analysis, there was a significant main effect of time both post-intervention and at follow-up in the expected direction (i.e., improvement of clients’ condition) on all outcome measures (see Table 2).

**TABLE 2:** Effects of music therapy with or without resonance frequency breathing or listening homework.

	Baseline (T0)		Post-intervention (T1, 6 weeks)								Follow-up (T2, 6 months)							
	No	Yes	No	Yes	Crude			Adjusted			No	Yes	Crude			Adjusted		
	Mean (SD)	Mean (SD)	Mean (SD)	Mean (SD)	B (SE)	95% CI	*p*-value	B (SE)	95% CI	*p*-value	Mean (SD)	Mean (SD)	B (SE)	95% CI	*p*-value	B (SE)	95% CI	*p*-value
MADRS RFB	22.80 (7.38)	26.71 (6.18)	15.87 (8.02)	14.38 (9.40)	−3.12 (1.90)	−6.88 to 0.64	0.103	−3.93 (1.99)	−7.85 to −0.01	0.049*	16.34 (10.37)	14.06 (8.85)	−3.98 (1.92)	−7.77 to −0.19	0.040*	−4.77 (2.00)	−8.72 to −0.82	0.018*
MADRS LH	25.32 (6.95)	24.22 (7.17)	16.13 (8.30)	14.09 (9.16)	−1.36 (1.90)	−5.11 to 2.39	0.476	−1.07 (2.09)	−5.19 to 3.06	0.611	16.58 (10.67)	13.69 (8.28)	−2.04 (1.92)	−5.82 to 1.74	0.289	−1.76 (2.10)	−5.91 to 2.39	0.404
HADS RFB	9.63 (4.22)	11.57 (3.30)	8.20 (3.85)	7.56 (4.16)	−1.79 (0.80)	−3.37 to −0.21	0.027*	−2.30 (0.85)	−3.97 to −0.62	0.008**	7.79 (4.30)	7.41 (3.99)	−1.57 (0.81)	−3.17 to 0.03	0.054	−2.07 (0.86)	−3.76 to −0.38	0.017*
HADS LH	11.41 (3.88)	9.83 (3.79)	8.26 (3.99)	7.48 (4.03)	0.21 (0.80)	−1.38 to 1.79	0.798	0.59 (0.85)	−1.08 to 2.27	0.486	7.90 (4.22)	7.28 (4.03)	0.39 (0.81)	−1.20 to 1.99	0.625	0.78 (0.85)	−0.91 to 2.46	0.364
RAND36 MCS RFB	−5.51 (2.53)	−6.33 (2.48)	−3.14 (3.40)	−2.08 (3.75)	1.52 (0.68)	0.18 to 2.86	0.027*	1.69 (0.71)	0.29 to 3.09	0.018*	−3.04 (3.97)	−1.74 (3.49)	1.75 (0.69)	0.40 to 3.10	0.012*	1.92 (0.71)	0.52 to 3.33	0.008**
RAND36 MCS LH	−6.12 (2.51)	−5.74 (2.55)	−2.86 (3.32)	−2.31 (3.88)	0.40 (0.68)	−0.94 to 1.74	0.552	0.45 (0.68)	−0.89 to 1.79	0.512	−2.62 (3.89)	−2.07 (3.64)	0.39 (0.68)	−0.96 to 1.74	0.572	0.43 (0.68)	−0.92 to 1.78	0.535
RAND36 PCS RFB	−3.59 (2.97)	−3.71 (2.72)	−2.18 (3.17)	−1.05 (3.24)	1.38 (0.55)	0.31 to 2.46	0.012*	1.41 (0.55)	0.32 to 2.50	0.011*	−2.11 (3.73)	−0.91 (3.03)	1.44 (0.55)	0.36 to 2.53	0.010**	1.47 (0.56)	0.37 to 2.57	0.009**
RAND36 PCS LH	−4.07 (2.91)	−3.26 (2.73)	−2.05 (3.44)	−1.13 (3.01)	0.40 (0.55)	−0.68 to 1.48	0.463	0.32 (0.56)	−0.79 to 1.43	0.571	−1.89 (3.59)	−1.05 (3.20)	0.30 (0.55)	−0.79 to 1.38	0.587	0.21 (0.57)	−0.90 to 1.33	0.704
GAF RFB	60.20 (7.77)	56.69 (8.23)	73.40 (12.06)	74.26 (13.06)	2.76 (2.42)	−2.03 to 7.54	0.257	3.31 (2.49)	−1.61 to 8.22	0.186	73.97 (14.06)	77.00 (12.90)	5.00 (2.45)	0.17 to 9.82	0.042*	5.53 (2.51)	0.58 to 10.48	0.029*
GAF LH	57.71 (8.82)	59.14 (7.50)	71.87 (12.15)	75.73 (12.74)	2.84 (2.42)	−1.94 to 7.61	0.243	2.54 (2.62)	−2.63 to 7.72	0.333	73.00 (14.94)	78.12 (11.45)	3.82 (2.44)	−0.99 to 8.63	0.119	3.54 (2.64)	−1.66 to 8.75	0.181

This table shows observed means and SDs for the ITT sample, and estimates of treatment effects (RFB_yes_ vs. RFB_no_; LH_yes_ vs. LH_no_) from linear-effects models. Results of the crude (corrected for baseline value and repeated measures) and adjusted (corrected for baseline value, repeated measures, client age, client gender, medication, and assigned therapist) treatment efficacy analyses for the ITT population. No and Yes indicate absence or presence of each factor (RFB and LH). Total *n*: 70; RFB_no_*n*: 35; RFB_yes_*n*: 35; LH_no_*n*: 34; LH_yes_*n*: 36. *p < 0.05; **p < 0.01.

**FIGURE 2:**
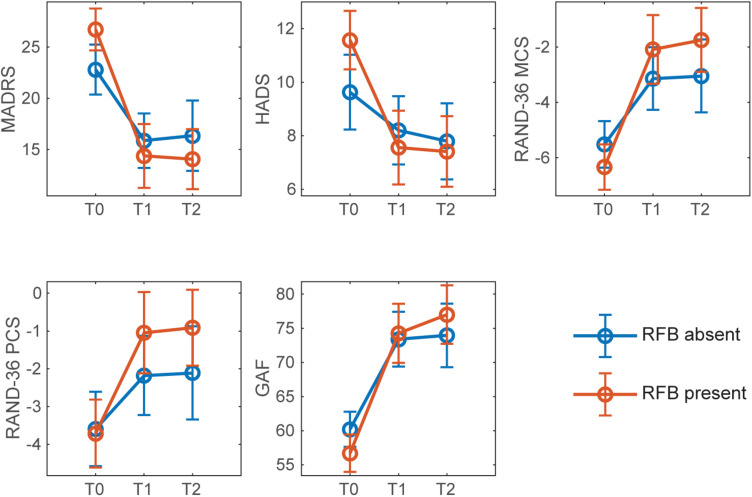
Mean scores of continuous outcome for presence and absence of RFB across timepoints. Error bars denote confidence intervals at 95%. T0: baseline; T1: post-intervention (6 weeks after the beginning of the intervention); T2: follow-up (6 months after the beginning of the intervention).

**FIGURE 3:**
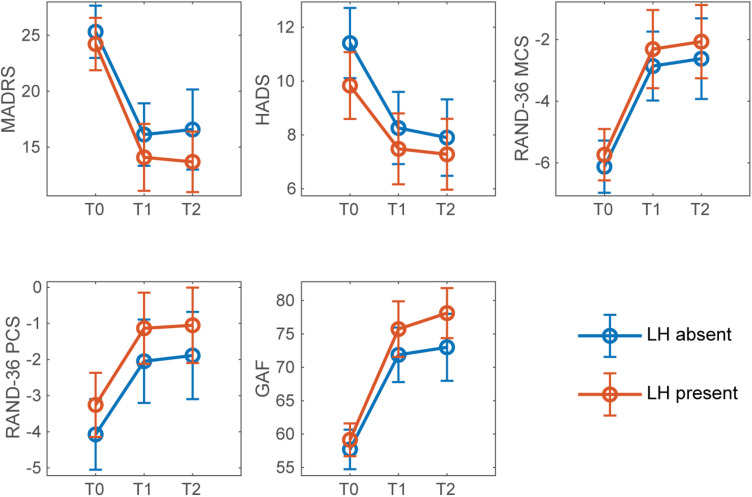
Mean outcome measure scores for presence and absence of LH across timepoints. Error bars denote confidence intervals at 95%. T0: baseline; T1: post-intervention (6 weeks after the beginning of the intervention); T2: follow-up (6 months after the beginning of the intervention).

Figures 2, 3 show mean outcome scores across time points, separately for presence and absence of RFB and LH. An overall improvement over time for all secondary measures can be observed, regardless of condition.

Table 2 shows the results of crude and adjusted treatment efficacy analyses post-intervention and at follow-up. The crude treatment efficacy analyses revealed significant differences between RFB_yes_ and RFB_no_ for all outcome measures, in all cases favouring RFB_yes_. The differences between most outcome measures both post-intervention and at follow-up reached statistical significance. Regarding LH, although the results for most outcome measures favoured LH_yes_ (with the exception of HADS), none of them reached significance. Adjusted treatment efficacy analyses yielded similar results to those obtained in the crude analyses, except that the adjusted analyses for RFB reached significance at both time points for all outcome measures. Potential interactions between RFB and LH were examined by subsequently adding an RFB x LH interaction. This factor interaction, however, did not yield significance at any time point for any outcome measure, neither in the crude nor in the adjusted analysis.

Crude and adjusted overall treatment effect over time and resulting effect sizes are presented in Table 3. According to the crude treatment efficacy analysis, the overall effect of treatment for RFB was significant for all measures except GAF, with RFB_yes_ clients invariably improving more than RFB_no_ clients. The adjusted treatment efficacy analysis yielded similar results, except for two differences. First, while the overall effect of treatment for GAF did not reach significance for RFB in the crude analysis, all outcome measures yielded significant differences for RFB in the adjusted analysis. Second, differences between RFB_yes_ and RFB_no_ increased after covariate adjustment of the treatment efficacy analysis, especially for MADRS.

**TABLE 3:** Effect sizes of music therapy with or without resonance frequency breathing for continuous outcomes.

	Estimates from crude analysis			Estimates from adjusted analyses		
	Raw difference B (SE)	*p*	Effect size *d* (95% CI)	Raw difference B (SE)	*p*	Effect size *d* (95% CI)
MADRS	−3.55 (1.53)	0.02*	0.50 (0.07 to 0.93)	−4.35 (1.64)	0.009**	0.62 (0.16 to 1.08)
HADS	−1.68 (0.67)	0.01*	0.43 (0.09 to 0.77)	−2.18 (0.73)	0.003**	0.56 (0.19 to 0.93)
RAND-36 MCS	1.63 (0.56)	0.004**	0.65 (0.21 to 1.09)	1.81 (0.60)	0.003**	0.72 (0.25 to 1.18)
RAND-36 PCS	1.41 (0.46)	0.003**	0.50 (0.18 to 0.82)	1.44 (0.47)	0.002**	0.51 (0.18 to 0.84)
GAF	3.87 (2.00)	0.05	0.48 (−0.01 to 0.96)	4.41 (2.08)	0.04*	0.54 (0.04 to 1.05)

Crude and adjusted overall treatment effect over time (B), *p*-values, and effect sizes (*d*) for primary and secondary measures (RFB factor). Crude and adjusted analyses are based on LME models. Effect sizes were calculated by dividing raw differences by pooled baseline SDs. *p < 0.05; **p < 0.01

### Primary Outcome

Montgomery-Åsberg Depression Rating Scale scores decreased in all conditions post-intervention, as shown in Figures 2, 3. An overall improvement in MADRS from moderate (20-34 points) to mild depression (7-19 points) can be observed for all conditions. Overall, the post-intervention remission rate (defined as MADRS ≤ 9) was 31%, and the post-intervention response rate (defined as a MADRS reduction of 50% or more) was 39%.

Regarding treatment effect post-intervention and follow-up for the RFB factor (see Table 2), there was no significant difference between conditions in MADRS (*p* = 0.103) post-intervention (6 weeks). However, at follow-up (6 months), the decrease in MADRS score was significantly larger in the RFB_yes_ condition than in the RFB_no_ condition (*p* = 0.04). No significant differences were found between the LH factor levels, neither at post-intervention (*p* = 0.485) nor follow-up (*p* = 0.297).

Overall treatment effect analyses [3] (see Table 3) showed a significantly higher decrease in MADRS for RFB_yes_ than for RFB_no_ (Crude B [SE] = −3.55 [1.53], p = 0.02^∗^). These differences increased after adjustment for potential confounders (Adjusted B [SE] = −4.35 [1.64], *p* = 0.009^∗∗^). No significant differences were found between LH factor levels (Crude B [SE] = −1.70 [1.53], *p* = 0.27; adjusted B [SE] = −1.42 [1.76], *p* = 0.42). Medium effect sizes for RFB were observed in both crude and adjusted analysis, although they were higher in the adjusted analysis (*d* [95% CI] = 0.62 [0.16−1.08]) than in the crude analysis (*d* [95% CI] = 0.50 [0.07−0.93]). For LH, small effect sizes (Crude *d* [95% CI] = 0.24 [−0.19−0.67]; Adjusted *d* [95% CI] = 0.20 [−0.29−0.70]) were observed.

Results for dichotomous variables are presented in Table 4. There were fewer dropouts in RFB_yes_ compared to RFB_no_ but the odds ratio was not significant. MADRS response rates were significantly greater in RFB_yes_ (*p* < 0.05) post-intervention (6 weeks), but were not significant at follow-up (6 months). A risk difference of 0.26 and NNT of 3.9 were observed, favouring RFB_yes_ condition. There were no significant differences between the LH factor levels in any of the dichotomous variables.

**TABLE 4:** Attrition and response rates in 70 participants randomised to music therapy with or without resonance frequency breathing or listening homework.

	Post-intervention (T1, 6 weeks)				Follow-up (T2, 6 months)			
Outcome	n/N (%) *No*	n/N (%) *Yes*	Odds Ratio (95% CI)	*p*	n/N (%) *No*	n/N (%) *Yes*	Odds Ratio (95% CI)	*p*
RFB Leaving the study early	5/35 (14.29)	1/35 (2.86)	0.18 (0.02–1.60)	0.20	6/35 (17.14)	1/35 (2.86)	0.14 (0.01–1.25)	0.11
LH Leaving the study early	3/34 (8.82)	3/36 (8.33)	1 (0.19–5.34)	1	3/34 (8.82)	4/36 (11.11)	1.38 (0.28–6.68)	1
RFB Response^a^	9/35 (25.71)	18/35 (51.43)	3.06 (1.12–8.37)	0.049*	9/35 (25.71)	16/35 (45.71)	2.43 (0.89–6.67)	0.13
LH Response^a^	11/34 (32.35)	16/36 (44.44)	1.67 (0.63–4.43)	0.33	12/34 (35.29)	13/36 (36.11)	1.04 (0.39–2.76)	1

*Results of dichotomous variables for primary outcome (MADRS). Missing information was treated as no change (intention-to-treat analysis). *N* = 70. RFB_no_*n* = 35;*

*RFB_yes_*n* = 35; LH_no_*n* = 34; LH_yes_*n* = 36.*

^*a*^ Response was defined as a reduction in MADRS scores of 50% or greater.

*p < 0.05.

### Secondary Outcomes

The crude treatment efficacy analyses resulted in a significant improvement in secondary measures for RFB_yes_ either at follow-up, post-intervention, or both time points (see Table 2). HADS scores decreased in all conditions during the intervention. In regards to RFB, there was a significant difference between conditions in HADS (*p* = 0.027) post-intervention. At follow-up, differences did not reach significance (*p* = 0.054). No significant differences were found between the LH factor levels neither at post-intervention nor follow-up; similar results regarding LH were observed for the other three secondary measures (RAND-36 MCS, RAND-36 PSY and GAF). Adjusted treatment effect analyses yielded comparable results, albeit of higher significance; this was also observed for the rest of the secondary outcomes. Also, in the adjusted analysis there was a significant difference in HADS (*p* = 0.017) between RFB_no_ and RFB_yes_ at follow-up.

For all conditions, both RAND-36 MCS and RAND-36 PCS decreased during intervention. For RAND-36 MCS, RFB results showed a significant difference between conditions, both post-intervention (*p* = 0.027) and at follow-up (*p* = 0.012), in favour of RFB_yes_. Significant differences were also observed between RFB_yes_ and RFB_no_ for RAND-36 PCS, both post-intervention (*p* = 0.012) and at follow-up (*p* = 0.01).

All conditions exhibited a decrease in GAF scores. There was no significant difference between conditions in GAF for RFB (*p* = 0.257) post-intervention (6 weeks). However, GAF scores at follow-up (6 months) were significantly higher in RFB_yes_ than in RFB_no_ (*p* = 0.042).

Regarding the overall crude treatment effect of secondary measures (see Table 3), we observed significant differences between RFB conditions for HADS (B [SE]: −1.68 [0.67], *p* = 0.01^∗^), RAND-36 MCS (B [SE]: 1.63 [0.56], *p* = 0.004^∗∗^) and RAND-36 PCS (B [SE]: 1.41 [0.46], *p* = 0.003^∗∗^). No significant differences in GAF were observed for RFB. With respect to LH, overall treatment effect analyses did not yield significant differences for any of the secondary measures. The adjusted overall treatment effect analysis yielded similar findings, although the differences between RFB_yes_ and RFB_no_ were larger, and GAF results reached significance. Crude effect sizes for RFB were medium or above medium for RAND-36 MCS and RAND-36 PCS, and close to medium for HADS and GAF. Adjusted effect sizes for RFB were close to large for RAND-36 MCS and above medium for HADS, RAND-36 PCS, and GAF. Regarding the LH factor, crude and adjusted effect sizes were trivial (d ≤ 0.2) for all outcome measures except GAF, which yielded higher effect sizes (Crude *d* [95% CI] = 0.41 [−0.07−0.89], Adjusted *d* [95% CI] = 0.37 [−0.17−0.92]).

### Sensitivity Analyses

Two sensitivity analyses were conducted. The first assumed no change in MADRS scores for missing observations, thus providing a conservative estimate for dropouts. Overall treatment effect for RFB was still significant in both crude (*p* = 0.003^∗∗^) and adjusted analysis (*p* = 0.002^∗∗^). Furthermore, a per-protocol analysis reclassified three clients from LH_yes_ to LH_no_, as they did not engage in any form of listening homework. There were still no significant differences between the LH factor levels in any of the outcome measures. Reclassification of clients for the RFB factor was not needed, since they all followed protocol.

### Adverse Events and Reasons for Drop-Out

Adverse events were rare, transient, and mostly unrelated to the trial interventions. Two participants (one IIMT + RFB, one IIMT + LH) experienced a worsening of problems (sleep problems) following a change in their medication. One (IIMT) had to stop therapy due to a pre-existing comorbid condition which necessitated surgery and subsequent recovery time. One (IIMT + LH) stopped therapy because a therapeutic alliance (agreement on goals and methods of therapy) could not be established. Finally, two participants (one IIMT, one IIMT + LH) stopped therapy due to scheduling issues.

## Discussion

In this study, we investigated whether a music therapy model called IIMT could be further enhanced by introducing additional components known to favour emotional processing and/or stress regulation (listening homework – LH, and resonance frequency breathing – RFB). In line with our previous RCT (Erkkilä et al., 2011), we found that 12 bi-weekly sessions of music therapy were able to significantly improve MADRS scores in all four conditions. Furthermore, our results indicate that IIMT can indeed be further enhanced, at least with RFB. More specifically, the overall effect of treatment for RFB was statistically significant for all measures except GAF, with RFB clients consistently improving more than non-RFB clients (see Table 3). We also observed significant differences in all outcome measures—either post-intervention, at follow-up, or both—favouring clients allocated to RFB (see Table 2). In contrast, the LH factor did not yield significant differences in any of our analyses. However, for all outcome measures besides HADS, the observed changes did favour LH_yes_. In sum, these results strongly support the hypothesis of RFB as an enhancer of therapeutic outcome and speak for its inclusion in music therapy, and possibly in other forms of psychotherapy.

Interestingly, for RFB_yes_, the treatment effect at T2 was larger than at T1 for all outcome measures except HADS, and the mean improvement in RFB_yes_ was monotonic (i.e., continued to increase between post-intervention and follow-up). Although we did not monitor whether clients kept using RFB on their own after the end of therapy, it is possible that an independent practice of RFB might have contributed to maintaining and reinforcing these positive outcomes.

In terms of clinical significance, the addition of RFB resulted in a near doubling of the MADRS post-intervention response rate, which went from 26% (RFB_no_) to 51% (RFB_yes_). To put these results into perspective, in our previous depression study (consisting of 20 bi-weekly sessions of music therapy without enhancers), the post-intervention response rate was 45% (Erkkilä et al., 2011). It is not surprising that 12 sessions of music therapy without RFB would result in a lower response rate than 20 sessions. However, the truly interesting finding is that, in terms of response rate, 12 sessions of music therapy with RFB were equivalent to 20 sessions without enhancers. Although this is a *post hoc* comparison of two different trials, it suggests that integrating RBF into music therapy might allow similar results to be achieved with fewer sessions.

These results point to the existence of qualities specific to RFB and music therapy which, when combined, can create a synergy effect. In our experience (Brabant and Erkkilä, 2018), clients who are starting their therapy sessions with RFB tend to have deeper and more productive sessions, which we attribute to RFB’s ability to rebalance the autonomic nervous system, reduce stress, and increase emotional resilience (Goessl et al., 2017). As to improvisational music therapy, three of its unique characteristics are to offer a non-verbal way of expressing emotions, to provide an absorbing experience anchored in the present, and to allow the emergence of unconscious material (MacDonald and Wilson, 2014). Thus, it stands to reason that combining the two methods would greatly facilitate the emergence of themes and emotions that usually remain unexpressed, while making it easier for the client to face these emotions and process them.

On a more general level, these findings highlight the benefits that can be derived from integrating RFB into an existing therapy method, instead of simply using it as an adjunct or complementary exercise, as is still largely the case when RFB or HRVB are being used. While searching the literature, we only found a few instances where such integration took place (e.g., Polak et al., 2015) or was being advocated (e.g., Gevirtz, 2020). Studies employing HRVB as a stand-alone intervention could serve as a baseline to determine the magnitude of possible synergy effects obtained in studies such as ours, by comparing effect sizes.

In contrast to RFB, our second added component (LH) did not yield any significant effect, in any of the analyses or comparisons that we performed. However, the changes observed at T1 and T2 were, nonetheless, always in favour of LH_yes_, except for HADS. In other words, the clients in the LH_yes_ condition benefited more from therapy than the clients in the LH_no_ condition. A more detailed analysis which is beyond the present paper will address the question whether listening duration correlated with clinical change. For such an analysis it will be important to separate extended, likely intentional listening from very short listening such as in searching for a piece.

Lastly, it should be noted that our results are in line with the existing evidence presented in the Introduction, regarding the positive effect of psychotherapy on comorbid anxiety (Weitz et al., 2018) and QoL (Kolovos et al., 2016). Interestingly, in this case, although the addition of RFB had a positive impact on both the physical and mental health component of QoL, the effect was more pronounced for physical health. We speculate that this was due to the nature of RFB and the regular practice thereof, which might have led to a sustained increase in autonomic flexibility and HRV, thus allowing clients to better regulate their stress levels in daily life and reduce unpleasant physical sensations.

## Limitations

The main limitations of this trial include limited sample size and lack of a no-treatment or placebo control group. Although the sample was large enough to detect a significant effect of breathing added to IIMT, it was not large enough to exclude a clinically meaningful effect of listening homework. Further research with a larger sample would be required to confirm or disconfirm any effects of this component. The sample was also restricted to a single site, so that conclusions generalising to other settings or world regions cannot be drawn with confidence. Second, the study did not use a no-treatment or placebo control group. However, robust effects of IIMT compared to standard care were already demonstrated in the previous study on which the present study was built (Erkkilä et al., 2011).

An issue surrounding LH is the absence of prior studies making use of this specific activity, which might have led to an incorrect estimation of the expected effect size. Although the use of homework has a long history in CBT, the kind of task given in CBT is arguably not directly comparable to what was required from the clients in the present trial. Thus, it is possible that our sample size was too small to detect a significant effect for the LH factor.

Another issue with LH might have been its possible inadequacy for the client population under investigation. Indeed, in contrast to RFB, LH was unsupervised, meaning that clients were free to perform the task or not, which led to lower task adherence compared to RFB. This raises the question of whether clients presenting with symptoms of depression should be given voluntary and unsupervised tasks in between therapy sessions, since depression typically includes a lack of initiative.

Future studies would benefit from having a larger sample size for studying LH, and being multi-centre. Furthermore, the results presented here are purely outcome-oriented, meaning it is not possible at this point to explain the results by establishing a relationship between what happened during therapy and the observed affective or behavioural changes.

Lastly, one question that remains unanswered is the extent to which the enhancement effect achieved with RFB in music therapy could be generalised to the larger field of psychotherapy. Based on our results, we presume that other forms of therapy would similarly benefit from the inclusion of RFB, especially if their approach and principles are similar to the ones used in music therapy (e.g., being emotion-focused, experiential, and integrative). Should this be the case, it would open the door to shorter and more cost-effective forms of therapy.

## Data Availability Statement

The raw data supporting the conclusions of this article will be made available by the authors, without undue reservation.

## Ethics Statement

The authors assert that all procedures contributing to this work comply with the ethical standards of the relevant national and institutional committees on human experimentation and with the Helsinki Declaration of 1975, as revised in 2008. All procedures involving human subjects/patients were approved by the Ethical board of Central Finland health care district, 07/09/2017, ref.: 17 U/2017. Written informed consent was obtained from every participant.

## Author Contributions

JE did the project leadership, contribution to the study design, development and implementation of the clinical music therapy model, writing parts of abstract, introduction, methods and discussion, and finalizing the manuscript. OB did the contribution to the study design, development and implementation of the RFB component, and writing parts of the methods and discussion sections. MH did the development and implementation of the LH component, statistical analysis, and writing parts of the methods, results, and discussion sections. AM did the statistical analysis, writing parts of the methods and results sections. EA-R developed and implemented the clinical music therapy model, wrote parts of the intervention, and commented the manuscript. NS did the development of LH component and implementation of the RFB component, helping to revise the methods and discussion section. SS did the contribution to the study design, helping to draft the results section and revise the manuscript. CG did the contribution to the study design, randomisation procedure, supervision of statistical analyses and revision of the manuscript text. All authors contributed to the article and approved the submitted version.

## Conflict of Interest

The authors declare that the research was conducted in the absence of any commercial or financial relationships that could be construed as a potential conflict of interest.
