# Supplementary material for: Music Therapy for Depression Enhanced With Listening Homework and Slow Paced Breathing: A Randomised Controlled Trial
Source: Front Psychol. 2021 Feb 16;12:613821. doi: 10.3389/fpsyg.2021.613821 (PMC7920974; doi:10.3389/fpsyg.2021.613821)
Supplement: Supplementary file 1 [file Data_Sheet_1.pdf]

Appendix

Changes to protocol article

Intention-to-treat approach

- Before: Modified intention-to-treat (mITT) analysis, which excludes from the analysis any subjects who drop out before the end of the intervention
- After: Intention-to-treat (ITT) analysis, meaning that the data from all subjects is used for the analysis, including those who dropped out before the end of intervention.
- Rationale: It was decided to go from a to a more accurate approach, which requires participants who strayed from the protocol to still be kept in the analysis.

Sample size calculation

- Before: Power calculation was based on t-test.
- After: Power calculation is based on a mixed model.
- Rationale: The power analysis was changed to reflect the type of statistical test that is used in the main analysis.

Rating of current depression severity

- Before: Rating was part of the analysis.
- After: Rating will be presented in future papers.
- Rationale: The scope of the present paper is to assess therapeutic outcome using predefined timepoints pre and post intervention (baseline, after intervention, after follow-up). Current depression severity was collected during the intervention and for each session. As such, it will be used alongside other measures collected during the intervention (movement, musical data, HRV) and will investigate the change of depression severity across therapy sessions.

Treatment of missing data

- Before: Clients who dropped out before the second measurement (T1) will be excluded from the analysis.
- After: Clients who dropped out before T1 are included in the analysis.
- Rationale: Repeated measures model allows to retain all clients in the analysis, including those for which only baseline scores are available. To follow an Intention-to-treat (ITT) approach, it was decided that clients with missing data will not be excluded.

RAND-36 detailed information

The inventory consists of 36 items ranging from 0-100 (higher scores denoting higher QoL) and representing 8 health-related scales from the aggregated items: physical functioning, role-physical, bodily pain, general health, vitality, social functioning, role-emotional, mental health.[1] Finnish population norms were used to standardize the scores of 8 scales relative to Finnish general public QoL.[2] Resulting standardized scores were grouped and aggregated into 2 summary measures (physical health and mental health), following the guidelines of Ware et al.[1]

Description of repeated-measures linear mixed-effects model

Treatment efficacy analyses were conducted using a repeated measures linear mixed model, which was corrected for baseline value by removing the treatment terms and for repeated measures by adding a random intercept term grouped by client.[3] The utilized overall treatment effect formula –equation 2c in [3] – is represented in Wilkinson-Rogers [4] notation as follows:

Outcome ~ 1 + Time + RFB:Time + LH:Time + (1 | id).

In this formula, Outcome includes the values of all three measurements of the outcome variable, Time describes the time points (0: baseline; 1: after intervention; 2: after follow-up) corresponding to each measurement, RFB and LH are treatment variables (false: treatment is absent; true: treatment is present), and id is a value between 1 and 64 that is used as client identifier. Time was treated as a categorical variable in order to allow for non-linear development of outcomes. The treatment effects after intervention and follow-up were obtained via the following formula (equation 2d in [3]):

Outcome ~ 1 + time_1 + time_2 + time_1:RFB + time_2:RFB + time_1:LH + time_2:LH + (1 | id),

where time_1 and time_2 are dummy variables representing time after intervention and after follow-up, respectively. The model was computed with the *fitlme* function of Matlab 2019b (Mathworks, Natick, USA), Statistics and Machine Learning Toolbox.

References

1 Ware JE, Kosinski M, Keller SD. *SF-36 physical and mental health summary scales: A user's manual*. Health Assessment Lab, 1994.
2 Aalto A, Aro AR, Teperi J. RAND-36 terveyteen liittyvän elämänlaadun mittarina*. Stakes Tutkimuksia* 1999; **101**.
3 Twisk J, Bosman L, Hoekstra T, Rijnhart J, Welten M, Heymans M. Different ways to estimate treatment effects in randomised controlled trials*. Contemporary clinical trials communications* 2018; **10**: 80-5. doi:https://doi.org/10.1016/j.conctc.2018.03.008.
4 Wilkinson GN, Rogers CE. Symbolic description of factorial models for analysis of variance*. Journal of the Royal Statistical Society: Series C (Applied Statistics)* 1973; **22**: 392-9. doi:https://doi.org/10.2307/2346786.
